# Ingol and Ingenol-Type Diterpenes from *Euphorbia trigona* Miller with Keratinocyte Inhibitory Activity

**DOI:** 10.3390/plants10061206

**Published:** 2021-06-14

**Authors:** Reham Hammadi, Norbert Kúsz, Csilla Zsuzsanna Dávid, Zoltán Behány, László Papp, Lajos Kemény, Judit Hohmann, Lóránt Lakatos, Andrea Vasas

**Affiliations:** 1 Department of Pharmacognosy, Interdisciplinary Excellence Centre, University of Szeged, Eötvös u. 6, 6720 Szeged, Hungary; reham.hammadi@pharmacognosy.hu (R.H.); kusznorbert@gmail.com (N.K.); davidzsuzsanna88@gmail.com (C.Z.D.); hohmann.judit@szte.hu (J.H.); 2 Department of Dermatology and Allergology, University of Szeged, Korányi fasor 6, 6720 Szeged, Hungary; behany.zoltan@med.u-szeged.hu (Z.B.); kemeny.lajos@med.u-szeged.hu (L.K.); 3 Botanical Garden, Eötvös Loránd University, Illés u. 25, 1083 Budapest, Hungary; papplaca@gmail.com; 4 Interdisciplinary Centre of Natural Products, University of Szeged, Eötvös u. 6, 6720 Szeged, Hungary; 5 Photo- and Chronobiology Group Eötvös Loránd Research Network (ELKH), Institute of Plant Biology, Biological Research Center Szeged, Temesvári krt. 62, 6726 Szeged, Hungary

**Keywords:** Euphorbiaceae, *Euphorbia trigona*, ingol esters, ingenol diterpenes, keratinocyte, cytotoxicity, actinic keratosis

## Abstract

Ingenol mebutate, isolated from *Euphorbia peplus*, is an ingenane-type diterpenoid, primarily used for the topical treatment of actinic keratosis, a premalignant skin condition. The aim of our work was to investigate other *Euphorbia* species to find structurally similar diterpenes that can be used as alternatives to ingenol mebutate. Pharmacological investigation of *Euphorbia candelabrum*, *Euphorbia cotinifolia*, *Euphorbia ramipressa*, and *Euphorbia trigona* revealed the potent keratinocyte (HPV-Ker cell line) inhibitory activity of these spurge species. From the methanolic extract of the aerial parts of *Euphorbia trigona* Miller, the most active species, five ingol (**1**–**5**) and four ingenane-type diterpenoids (**6**–**9**) were isolated by various chromatographic separation techniques, including open column chromatography, vacuum liquid chromatography, thin-layer chromatography, and high-performance liquid chromatography. The structures of the compounds were determined by NMR spectroscopic analysis and by comparison of the assignations with the literature data. The cytotoxic activity of the compounds against keratinocytes was tested in vitro by using ingenol mebutate as a positive control. Among the isolated compounds, two ingenane derivatives (**6** and **7**) exhibited remarkably stronger cytotoxic activity (IC_50_ values 0.39 μM and 0.32 μM, respectively) on keratinocytes than ingenol mebutate (IC_50_ value 0.84 μM). These compounds could serve as starting materials for further investigations to find alternatives to Picato^®^ (with active substance ingenol mebutate), which was withdrawn from marketing authorization in the European Union.

## 1. Introduction

Actinic keratosis (AK) is a skin disorder with erythematous plaques that are frequently found on sun-damaged skin [1]. AK is more likely to occur in men than women and is more prevalent in older adults [2,3]. The main causative agent of AK is excessive UV radiation, which can lead to destructive inflammatory processes, and ultimately to substantial structural damage to cell DNA and membrane lipids [4].

AK is considered to be an epidermal dysplasia, in which keratinocytes in the stratum basale and stratum spinosum have lost polarity, and appear disordered with pleomorphic nuclei. The stratum corneum shows parakeratosis and hyperkeratosis, most probably due to abnormal keratinocyte development [5]. In many AK patients, inflammatory infiltration of lymphocytes and plasma cells in the dermis can also be observed [6]. Although spontaneous regression of AK is very common (>50%) [7], the condition must be taken seriously, since it can develop into malignant lesions, such as squamous cell carcinoma (SCC), the second most common form of skin cancer, and basal cell carcinoma (BCC). These conditions predominantly affect light-skinned populations [6]. Treatment options for AK include destructive therapies (e.g., surgery, cryotherapy, dermabrasion, and photodynamic therapy (PDT)), topical medications (e.g., topical fluorouracil, imiquimod, ingenol mebutate, and diclofenac), and field ablation treatments (chemical peels and laser resurfacing) [8]. Self-directed treatments are preferred by patients, but are time-consuming, as they need to be applied for weeks or months before producing clinical results. Although many options are available, the search for new and more effective agents of AK is in progress.

Ingenol mebutate (Picato^®^) is the active ingredient of the sap of *Euphorbia peplus* [9,10], a plant that has long been used in Australian folk medicine for the treatment of skin cancers. It is offered with a short treatment schedule (2–3 days), providing an effective and sustained clearance of AK lesions with a predictable onset and short duration of local skin responses [11]. Ingenol mebutate acts on keratinocytes in two different ways: (1) induces the cell death of aberrant keratinocytes, and (2) induces a lesion-directed immune response that is mediated, at least partially, by the enzyme family protein kinase C (PKC) [11]. The ingenol-mebutate-induced cell death is mediated through the PKCδ/mitogen-activated protein kinase (MEK)/extracellular signal-regulated kinase (ERK) pathway. PKCδ is a key mediator in cell differentiation and the inhibition of proliferation in various tissues. Ingenol mebutate directly binds to PKCδ and leads to its phosphorylation, which activates MEK/ERK signaling, resulting in decreased viability and cell proliferation [12].

The topical application of ingenol mebutate causes rapid cell death via necrosis. Subsequently, the concentration of TNF-α and IL-8 promptly increases, which leads to the recruitment and infiltration of neutrophils into the inflamed region, followed by the activation of an apoptotic cell death [13]. Unfortunately, Picato is no longer authorized in the EU, as the European Medicinal Agency has concluded that the medicine may increase the risk of skin cancer and that its risks outweigh its benefits. Searching for new analogues of ingenol mebutate with a higher potency and a more favorable side effect spectrum seems to be a rational aim of phytochemical researchers.

Most of the plants belonging to the genus *Euphorbia* (family Euphorbiaceae) contain a milky irritant latex and accumulate different types of diterpenoid esters, characteristic of the family [14,15]. In recent decades, numerous pharmacological investigations into Euphorbiaceae plants focused on the authentication of their traditional use in cancerous conditions and confirmed the efficacy of various diterpenes as promising antitumor agents [15,16]. However, no publication can be found in the literature about the keratinocyte inhibitory activity of the *Euphorbia* species.

In a continuation of our work dealing with the isolation of biologically active diterpenoids, different extracts, prepared from *E. candelabrum* Trémaux ex Kotschy, *E. cotinifolia* (L.) Millsp., *E. ramipressa* Croisat, and *E. trigona* Mill., were tested for their keratinocyte inhibitory activities. *E. candelabrum* and *E. cotinifolia* were reported to contain ingenol derivatives [17]. Although *E. ramipressa* was not investigated from the phytochemical and pharmacological perspectives, as it belongs to the same section (*Euphorbia* sect. *Euphorbia*) as *E. candelabrum* and other ingenol derivative-containing species (e.g., *E. nivulia*, *E. antiquorum*, *E. kamerunica*), it is worthy of investigation [18]. Based on the results of the pharmacological assay, *E. trigona* Mill. was chosen for further examination. *E. trigona* is native to Central Africa, where it is commonly planted around villages as a ceremonial plant [19]. In Japan and the United States, it is cultivated as an indoor ornamental plant [20]. In the traditional medicinal use of *E. trigona*, usually, some drops of the latex are added to palm wine for the treatment of severe cases of constipation or epileptic attacks. It is also used as an arrow and fish poison [19]. The plant is also well recognised in India, as it has been used in Ayurveda to combat infections (e.g., in the urinary tract) and to alleviate the symptoms inflammation [21].

Previous studies have revealed the presence of ingol and ingenol esters in the latex of *E. trigona* that are responsible for its strong skin-irritating and pesticidal properties [20]; while lectins, another important class of phytoconstituents described from Euphorbia latices, have been reported to possess potent erythrocyte agglutinating ability [19]. The latex also contains compounds acting as antimicrobial agents in the urinary tract by attenuating the virulence of the pathogens and facilitating the elimination of them via stimulating the host’s immune system [21,22]. Interestingly, the extracts of *E. trigona* displayed immunostimulant activity as well [23].

Previously, ingol- and ingenane-type diterpenoids were isolated from the latex of *E. trigona* [20]. Moreover, the presence of triterpenoids was reported from the stems of the plant [24].

The aim of our work was to isolate the diterpenoids from the plant and to investigate their effects on keratinocytes in order to find potential lead compounds for the effective treatment of AK.

## 2. Results and Discussion

The main risk factor of induction of AK is UV irradiation. The UVB component of sunlight damages DNA, which might cause mutations in p53 and other tumor suppressor genes [25]. Therefore, as an in vitro model of AK, we used the HPV-Ker cell line which contains the E6 oncoprotein of the human papillomavirus, leading to the degradation of the p53 tumor suppressor [26].

**Figure 1:**
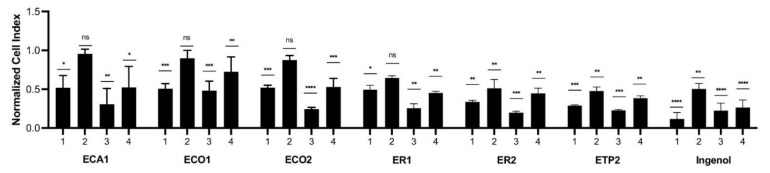
Inhibitory activity of different *Euphorbia* extracts against keratinocytes. ECA1: *E. candelabrum n*-hexane extract, ECO1: *E. cotinifolia n*-hexane extract, ECO2: *E. cotinifolia* CHCl_3_ extract, ER1: *E. ramipressa n*-hexane extract, ER2: *E. ramipressa* CHCl_3_ extract, ET1: *E. trigona n*-hexane extract, ET2: *E. trigona* CHCl_3_ extract; 1: 5 µg/mL, 24 h, 2: 0.5 µg/mL, 24 h, 3: 5 µg/mL, 48 h, 4: 0.5 µg/mL, 48 h; * *p* < 0.05; ** *p* < 0.01; *** *p* < 0.001; **** *p* < 0.0001.

The extracts, prepared from *E. candelabrum*, *E. cotinifolia*, *E. ramipressa*, and *E. trigona*, were tested at concentrations of 5 and 0.5 µg/mL for their inhibitory activity against keratinocytes (Figure 1). Ingenol mebutate administered at 5 µg/mL for 24 h exerted the strongest cytotoxic effect, and after a 48 h treatment, a lower cytotoxicity was measured. The treatment of keratinocytes with ingenol mebutate at a 0.5 µg/mL concentration resulted in a weaker, but still significant, cytotoxic effect after 24 h and, interestingly, after 48 h, the inhibitory activity was comparable to the treatment when 5 µg/mL was used for 48 h. The extracts with a 5 µg/mL concentration were applied for 24 h, and ET1 and ET2 had similar, but lower, cytotoxic activity than that of ingenol mebutate. Interestingly, the 48 h treatment of cells with 5 µg/mL extracts ECA1, ECO2, ER1, ER2, ET1, and ET2 showed a very similar cytotoxic property as ingenol mebutate. Cytotoxic activity was the lowest when the extracts were used at a 0.5 µg/mL concentration for 24 h, but in the case of ER2, ET1, and ET2, cytotoxic activity was significant compared to the control and quite similar to that of ingenol mebutate. After administration of the extracts at a 0.5 µg/mL concentration for 48 h, only ET1 displayed cytotoxicity comparable to that of ingenol mebutate. Based on these results, the *n*-hexane extract of *E. trigona* (ET1) could be considered the most promising one for further investigation.

Previous investigations into the plants resulted in the isolation of various diterpenoids, such as 3-*O*-propionyl-20-*O*-(*S*)-(2′-methyl)butyryl-ingenol, 20-*O*-isobutyryl-ingenol, 3-*O*-propionyl-20-*O*-isobutyryl-ingenol, and 3,20-*O*-di-isobutyryl-ingenol from the leaves of *E. cotinifolia* [27], and 17-acetoxyingenol 3 angelate 5,20-diacetate and 17-acetoxy-20-deoxyingenol 3-angelate, from the latex of *E. trigona* [20].

**Figure 2:**
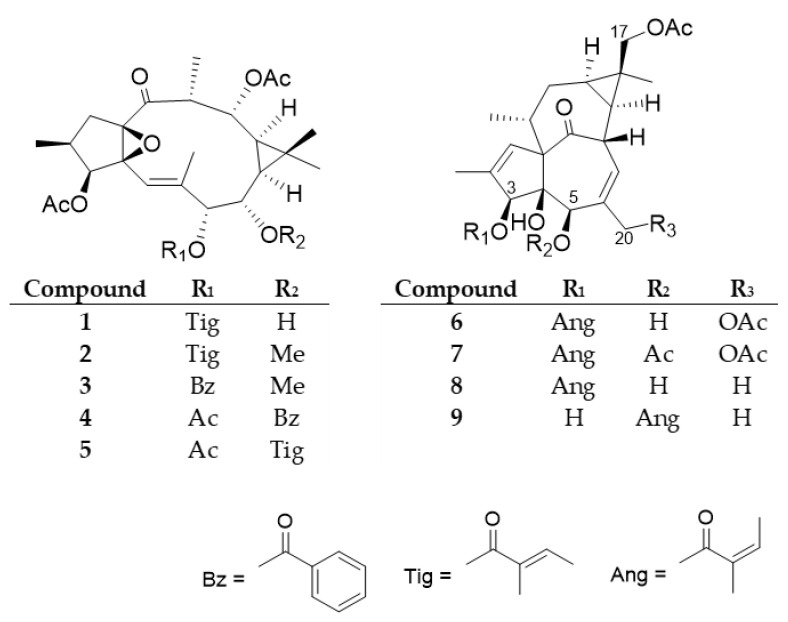
Structures of the isolated diterpenoids (**1**–**9**).

Among the investigated plants, the most active *E. trigona* was chosen for further phytochemical and pharmacological investigation. Chromatographic separation of the methanolic extract, prepared from the fresh aerial parts of *E. trigona* by open column chromatography using polyamide and vacuum liquid chromatography on silica gel, as well as by preparative TLC, and normal and reversed-phase HPLC, yielded nine diterpenoids (**1**–**9**), including five ingol (**1**–**5**) and four ingenol (**6**–**9**) esters (Figure 2).

The structures of the compounds were determined by comparison of their spectroscopic data to those of reported literature values (Supplemetary Materials Figures S1–S16). Diterpenoid esters **1**–**9** were described earlier from only a few plant species. Ingol 3,12-diacetate 7-tigliate (**1**) was previously isolated from *E. hermentiana* [28], 8-*O*-methyl-ingol 3,12-diacetate 7-tigliate (**2**) from *E. acrurensis* [29], *E. hermentiana* [28], and *E. kamerunica* [30], 8-*O*-methyl-ingol 3,12-diacetate 7-benzoate (**3**) from *E. antiquorum* [31], *E. hermentiana* [28], and *E. kamerunica* [30], ingol 3,7,12-triacetate 8-benzoate (**4**) from *E. antiquorum* [31], *E. nivulia* [16,32] *E. hermentiana* [28], and *E. kamerunica* [33], and ingol 3,7,12-triacetate 8-tigliate (**5**) from *E. antiquorum* [31], and *E. kamerunica* [33]. The ingenane-type compound 17-acetoxyingenol 3-angelate 20-acetate (**6**) was previously reported from *E. canariensis* [34] and *E. hermentiana* [35], 17-acetoxyingenol 3 angelate 5,20-diacetate (**7**) from *E. hermentiana* [35], *E. kamerunica* [30], *E. royleana* [36], and *E. trigona* [20], 17-acetoxy-20-deoxyingenol 3-angelate (**8**) from *E. acrurensis* [29], *E. hermentiana* [35] and *E. trigona* [20], and 17-acetoxy-20-deoxyingenol 5-angelate (**9**) from *E. hermentiana* [35]. None of the compounds were evaluated for keratinocyte viability modifying activity.

According to the classification of Pax and Hoffmann, *E. antiquorum* and *E. royleana* belong to the Section Euphorbium Bentham, Subsection Diacanthium Boiss., and Series Trigonae Berger (V), while *E. canariensis*, *E. candelabrum*, and *E. kamerunica* belong to the Series Polygonae Berger (VI) of the same Section and Subsection [37]. Therefore, botanically they are very close to each other, and it is not surprising that they contain structurally similar diterpenoids, as was proven by Evans and Kinghorn [38]. Moreover, all the species are succulent and originate from Africa.

After the discovery of ingenol mebutate as an effective drug for the treatment of AK, a number of semisynthetic derivatives were produced or synthesized with full regiocontrol from ingenol [11,39].

**Figure 3:**
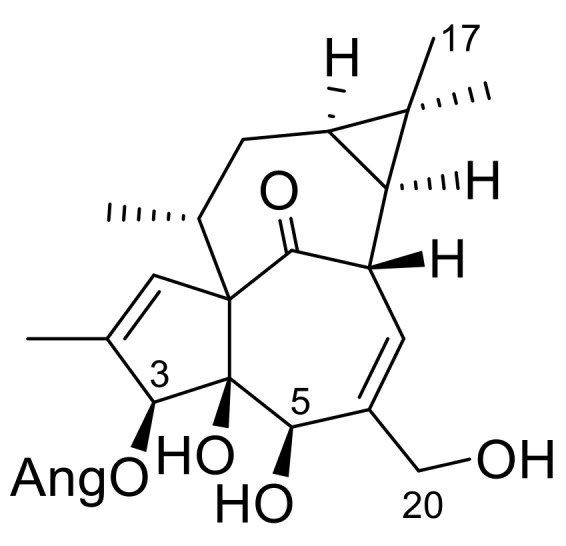
The structure of ingenol mebutate.

Since our compounds, especially the ingenol-derivatives, are structurally very similar to ingenol mebutate (Figure 3), we were interested in whether they have any effect on the viability of keratinocytes. The HPV-Ker cell line was treated with the isolated ingol- and ingenol-type diterpenoids with a concentration range of 5 × 10^−9^–5 × 10^−4^ M, the viability was measured with the xCELLigence System RTCA HT (Agilent Technologies, Santa Clara, CA, USA) instrument for 72 h, and data at 24 and 48 h were used for calculations (Table 1). IC_50_ values were calculated based on the sigmoidal dose–response formula.

**Figure 4:**
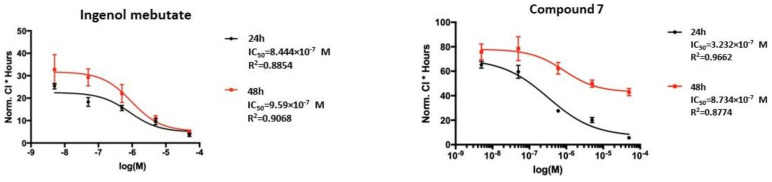
Real-time cell analysis (RTCA) measurement of CI (cell index) values of HPV-Ker cells treated with ingenol mebutate and compound **7**. Normalized CI * hours values were plotted as a function of concentration of the indicated diterpenoid (logM).

Ingenol mebutate, which was used as a positive control, showed IC_50_ values of 0.84 and 0.96 µM at 24 and 48 h of treatment (Figure 4), respectively. For the ingenol-type compound, 17-acetoxy-20-deoxyingenol 5-angelate (**9**), about one and two order of magnitude higher IC_50_ values, were recorded after 24 and 48 h of treatment (14.83 and 7.93 µM) than for ingenol mebutate (Supplementary Materials Figure S17). Strikingly, the IC_50_ values of the ingenane-type diterpenoids, 17-acetoxyingenol 3-angelate 20-acetate (**6**) and 17-acetoxyingenol 3 angelate 5,20-diacetate (**7**), were the same order of magnitude as ingenol mebutate (Figure 4 and Figure S1). Moreover, the IC_50_ values of **6** (0.39 µM and 0.32 µM) and **7** (0.32 µM and 0.87 µM) were slightly lower on the HPV-Ker cell line than that of ingenol mebutate (0.84 µM and 0.96 µM).

In the case of the ingol-type diterpenoids **1**, **2** and **4**+**5**, the measured IC_50_ values were one or two orders of magnitude higher than that of ingenol mebutate, except for **4**+**5** at 48 h (0.66 µM) (Table 1).

Based on the pharmacological results, structure–activity relationship (SAR) investigations could also be performed. The ingenol derivatives (**6**–**9**) are structurally close to ingenol mebutate. The main difference between ingenol mebutate and the isolated compounds is the presence of an acetoxy group at C-17 in **6**–**9** instead of a 17-methyl group in ingenol mebutate. In the less active 17-acetoxy-20-deoxyingenol 5-angelate (**9**), the angeloyl group at C-3, and hydroxy group at C-5, are replaced compared to ingenol mebutate and **6**–**8**; therefore, it was concluded that the presence of the angeloyl group at C-3 seems to be essential for the cytotoxic activity. Since compound **8** differs from ingenol mebutate in only the presence of an acetoxy group at C-17, and its activity was lower, the acetoxy group alone, presumably, is not able to increase the activity. In the case of the most active compounds **6** and **7**, one (at C-20 in **6**) or two (at C-5 and C-20 in **7**) additional acetyl groups are attached to the diterpenoid core; therefore, acetylation of the molecule results in increased cytotoxic activity. This is in accordance with the previously determined SAR statement that the carbonyl moieties of the ester groups are essential for the desired biological effects and the activation of PKC, which likely happens through interaction with Gly23 NH in the C1 domain [11]. Moreover, besides the activation of PKCδ, ingenol mebutate was found to reduce the expression of PKCα, which is the PKC isoform responsible for the promotion of cell survival [40]. Thus, further studies are required to evaluate the beneficial effect of our compounds that might result in more effective isoform-specific regulation.

In summary, among the ingol- and ingenol-type diterpenoids tested in our work, the ingenol-type 17-acetoxyingenol 3-angelate 20-acetate (**6**) and 17-acetoxyingenol 3 angelate 5,20-diacetate (**7**) showed stronger cytotoxic activity on keratinocytes after 24 and 48 h of administration than ingenol mebutate.

## 3. Materials and Methods

### 3.1. General Experimental Procedures

NMR spectra were recorded in MeOD and DMSO-*d*_6_ on a Bruker Avance DRX 500 spectrometer at 500 MHz (^1^H) and 125 MHz (^13^C). The signals of the deuterated solvents were chosen as references. The chemical shift values (*δ*) were given in ppm and coupling constants (*J*) in Hz. Two-dimensional (2D) experiments were performed with standard Bruker software. In the ^1^H–^1^H COSY, HSQC, and HMBC experiments, gradient-enhanced versions were used. Column chromatography (CC) was performed on polyamide (MP Biomedicals Germany GmbH). Normal phase vacuum liquid chromatography (VLC) was carried out on silica gel (Kieselgel 60 GF_254_, 15 µm, Merck, Darmstadt, Germany). Thin-layer chromatography was performed on Kieselgel 60 RP-18 F_254_ and Kieselgel 60 F_254_ (Merck). Spots on UV active silica gel were detected under UV light (245 nm and 336 nm) and made visible with vanillin sulphuric acid and heating at 105 °C for 2 min. The high-performance liquid chromatography (HPLC) separation was carried out on a Waters HPLC (Waters 600 controller, Waters 600 pump, and Waters 2998 photodiode array detector), using normal (LiChrospher Si 100 (250 × 4 mm, 5 μm, Merck, Darmstadt, Germany) and RP (LiChrospher RP-18 (5 μm, 250 × 4 mm, Merck, Darmstadt, Germany) columns. In the case of the gradient elution, the mobile phase consisted of cyclohexane (solvent A) and EtOAc (solvent B) with a flow rate of 1.5 mL/min at normal phase separation. The initial mobile phase composition was maintained at 80% solvent A for 1 min, changed linearly to 60% (1–10 min), then changed linearly to 80% (10–10.5 min) and held at 80% for 1 min (10.5–11.5 min), then followed by changing to 100% mobile phase A within 30 s (11.5–12 min) and kept 3 min (12–15 min) for the chromatography column equilibrium. In the case of the RP column, the mobile phase consists of MeOH (solvent A) and H_2_O (solvent B). The initial mobile phase composition was maintained at 80% solvent A for 1 min, changed linearly to 60% (1–10 min), then changed linearly to 80% (10–10.5 min) and held at 80% for 1 min (10.5–11.5 min), followed by a change to 100% mobile phase A within 30 s (11.5–12 min) and maintained for 3 min (12–15 min) for the chromatography column equilibrium. The flow rate was 1 mL/min and the injection volume was 25 μL. The data were acquired and processed with the Empower software.

All solvents used for CC were of at least analytical grade (VWR Ltd., Szeged, Hungary). Ultra-pure water was prepared with a Milli-Q water purification system (Merck, Darmstadt, Germany ).

### 3.2. Plant Materials

The aerial parts of *E. candelabrum*, *E. cotinifolia*, *E. ramipressa*, and *E. trigona* (100 g, each) were collected in the Botanical Garden of the University of Szeged and were identified by Anikó Németh (director of the Botanical Garden). The aerial parts (5.7 kg, fresh weight) of *E. trigona* Mill. were collected in June 2018, in the Botanical Garden of Eötvös Loránd University, Hungary, and were identified by one of the authors (L.P., Botanical Garden, Eötvös Loránd University, 1083 Budapest, Hungary). A voucher specimen (No. 891) was deposited at the Department of Pharmacognosy, University of Szeged, Szeged, Hungary.

### 3.3. Extraction and Isolation

The fresh plant materials of *E. candelabrum*, *E. cotinifolia*, *E. ramipressa*, and *E. trigona* (100 g, each) were extracted with methanol (3 × 500 mL, each) at room temperature. The extracts were concentrated in vacuo, and then dissolved in MeOH–H_2_O 1:1 (150 mL, each), and partitioned with *n*-hexane and CHCl_3_ (3 × 150 mL, each). The *n*-hexane and CHCl_3_ extracts were evaporated to dryness and used for pharmacological investigation.

The fresh aerial parts of *E. trigona* (5.7 kg) were blended and percolated with methanol (30 L) at room temperature. The methanol extract was concentrated in vacuo, and then dissolved in MeOH–H_2_O 1:1, and partitioned with *n*-hexane, CHCl_3_, EtOAc, and BuOH to obtain five fractions (*n*-hexane, CHCl_3_, EtOAc, BuOH, and aqueous residue). The fractions were monitored by normal phase thin-layer chromatography (TLC). After UV detection, the plates were sprayed with concentrated sulfuric acid and heated at 105 °C for 2 min. Based on the TLC determination, it could be observed that diterpenes were accumulated in the *n*-hexane fraction.

*n*-Hexane fraction (26.5 g) was separated by polyamide column chromatography with a MeOH–H_2_O gradient system (3:2, 4:1, and 1:0) to obtain three fractions (P1-3). Fraction P1 (10.0 g) was then separated by vacuum liquid chromatography (VLC) on silica gel with a gradient system of cyclohexane–EtOAc–EtOH (99:1:0–1:1:1; 500 mL of each). TLC determination and combination of the fractions afforded 20 main fractions (P1/1-20). Fractions P1/7 (164.8 mg), P1/8 (326.7 mg), P1/9 (160.9 mg), and P1/12 (96.8 mg) were purified by VLC on NP silica gel with a cyclohexane–EtOAc gradient solvent system (95:5:0–6:3:0.5) to yield combined fractions P1/7/1-4, P1/8/1-4, P1/9/1-4, and P1/12/1-6, respectively. Fractions P1/7/2 (89 mg), P1/8/3 (189 mg), P1/8/4 (161 mg), and P1/9/4 (82 mg) were further purified by preparative TLC on NP silica gel with toluene–acetone 8:2 as the mobile phase, and then by NP-HPLC with a gradient system of cyclohexane–EtOAc (flow rate 1.5 mL/min) to yield compounds: **4**+**5** (13.3 mg) from P1/7/2; **2** (45.7 mg) from P1/8/3; **3** (1.4 mg) from P1/8/4; **7** (6.8 mg); **9** (4.8 mg) from P1/9/4. Compound **1** (12.1 mg) was isolated from P1/9/4 by preparative TLC using toluene–acetone 8:2 as the mobile phase. Purification of P/1/12/4 (9.6 mg) by preparative TLC (NP, toluene–acetone 8:2) resulted in the isolation of compound **8** (1.9 mg). Fraction P/1/12/6 (12 mg) was separated by preparative TLC with toluene–acetone 7:3, and then by RP-HPLC with a gradient system of MeOH–H2O (flow rate 1 mL/min) to obtain compound **6** (6.5 mg).

### 3.4. Keratinocyte Inhibitory Activity Investigation

The immortalized human keratinocyte cell line (HPV-Ker) was cultured in Keratinocyte-SFM supplemented with 5 ng/mL of recombinant epidermal growth factor, 50 mg/mL of bovine pituitary extract, and antibiotic/antimycotic solution in a CO_2_ thermostat at 37 °C.

#### Viability Test

The effect of the extracts and the purified compounds on HPV-Ker cells was investigated by real-time monitoring with the xCELLigence System RTCA HT Instrument (ACEA Biosciences, San Diego, CA, USA). 8 × 10^3^ HPV-Ker cells were seeded in 96-well E-plates and keratinocytes were allowed to attach to the bottom of the wells and grow for 24 h. Cells were then treated with the indicated extract or substance for another 72 h. Real-time measurements of impedance were monitored every 15 min, then data at 24 and 48 h were used for calculations. The half-maximal inhibitory concentration (IC_50_) values for the compounds were calculated and GraphPad Prism 9.0 was used to plot results. All experiments were performed in duplicate, in three independent repeats.

## 4. Conclusions

The extracts of four *Euphorbia* species, namely *E. candelabrum*, *E. cotinifolia*, *E. ramipressa*, and *E. trigona*, were tested for their antiproliferative effect against keratinocytes. All species proved to be active. Among them, *E. trigona*, the most active, was chosen for further investigation. From the aerial parts of the plant, nine diterpenoids (**1**–**9**) were isolated, including five ingol (**1**–**5**) and four ingenol esters (**6**–**9**). 17-Acetoxyingenol 3-angelate 5,20-diacetate (**7**) and 17-acetoxy-20-deoxyingenol 3-angelate (**8**) were previously identified from the plant, while the others were described from other *Euphorbia* species. The diterpenoid profile of *E. trigona* was found to be very similar to that of *E. hermentiana*, confirming that these two species are chemotaxonomically closely related. The isolated compounds **1**–**9** have been tested for their keratinocyte inhibitory activity for the first time. Two ingenanes (**6** and **7**) demonstrated strong keratinocyte inhibitory activity in vitro which was comparable to that of ingenol mebutate, the drug used in the treatment of actinic keratosis. None of the ingol-type esters exerted considerable activity compared to ingenol mebutate.

The mechanism of action of ingenol mebutate is complex; it involves a combination of necrotic and immunostimulating effects. Previous studies established that the apoptotic properties of ingenol mebutate are based on the activation of PKCδ and its translocation from the cytoplasm into the nuclear membrane. Furthermore, the reduction in the expression of PKCα also plays a role in the effect of the compound. The interaction of ingenoids with PKC is critically dependent on their type of ester decoration and requires a combination of optimal hydrogen bonding and hydrophobic contacts for high potency [41]. To date, only limited information exists on the structure–activity relationships of ingenol esters as PKC ligands, and in the previous studies, the influence of 17-*O*-acyl groups present in **6** and **7** was not studied.

17-Acetoxyingenol 3-angelate 20-acetate (**6**) and 17-acetoxyingenol 3 angelate 5,20-diacetate (**7**) could serve as promising compounds in the search for novel therapeutic agents for actinic keratosis, as they are structurally very similar to ingenol mebutate and possess higher keratinocyte inhibitory activity. This higher activity is probably due to the acetoxy group(s), as it was previously observed that the carbonyl moieties of the ester groups are essential for the activation of PKC, and therefore, the biological effect. Based on the pharmacological screening study, the other investigated *Euphorbia* species (*E. candelabrum*, *E. cotinifolia*, and *E. ramipressa*) are also worthy of phytochemical and pharmacological investigation.

## Figures and Tables

**Table 1:** IC_50_ values (µM ± SD) of the ingol- and ingenane-type diterpenoids (**1**–**9**).

Compound	IC_50_ Value (μM)	
	24 h	48 h
**1**	14.19 ± 2.85	1.72 ± 0.14
**2**	17.29 ± 1.65	14.48 ± 3.78
**3**	inactive	
**4+5**	4.50 ± 0.93	0.66 ± 0.05
**6**	0.39 ± 0.09	0.32 ± 0.05
**7**	0.32 ± 0.02	0.87 ± 0.07
**8**	4.32 ± 0.92	-
**9**	14.83 ± 3.83	7.93 ± 1.71
**Ingenol mebutate**	0.84 ± 0.01	0.96 ± 0.03

## Supplementary Materials

The following are available online at https://www.mdpi.com/article/10.3390/plants10061206/s1. Figures S1–S16: ^1^H and ^13^C JMOD NMR spectra of compounds **1**–**9**. Figure S17: RTCA (real-time cell analysis) measurement of CI (cell index) values of HPV-Ker cells treated with compounds **1**, **2**, **4**+**5**, **6,** and **9**. Normalized CI * hours values were plotted as a function of concentration of the indicated diterpenoid (logM).
