# Supplementary material for: Ingol and Ingenol-Type Diterpenes from Euphorbia trigona Miller with Keratinocyte Inhibitory Activity
Source: Plants (Basel). 2021 Jun 14;10(6):1206. doi: 10.3390/plants10061206 (PMC8231945; doi:10.3390/plants10061206)
Supplement: Supplementary file 1 [file plants-10-01206-s001.zip › plants-1217085-supplementary.pdf]

SUPPORTING INFORMATION

Article

Ingol and Ingenol-type Diterpenes from *Euphorbia trigona* Mill. with Keratinocyte Inhibitory Activity

Reham Hammadi [1], Norbert Kúsz [1], Csilla Zsuzsanna Dávid [1], Zoltán Behány [2], László Papp [3], Lajos Kemény [2], Judit Hohmann [1,4], Lóránt Lakatos [2,*] and Andrea Vasas [1,*]

1 Department of Pharmacognosy, Interdisciplinary Excellence Centre, University of Szeged, 6720 Szeged, Eötvös u. 6., Hungary; reham.hammadi@pharmacognosy.hu, kusznorbert@gmail.com, davidzsuzsanna88@gmail.com, hohmann.judit@szte.hu, vasas.andrea@szte.hu
2 Department of Dermatology and Allergology University of Szeged, 6720 Szeged, Korányi fasor 6., Hungary; behany.zoltan@med.u-szeged.hu, kemeny.lajos@med.u-szeged.hu, lakatos.lorant@ brc.hu
3 Botanical Garden, Eötvös Loránd University, 1083 Budapest, Illés u. 25, Hungary; papplaca@gmail.com
4 Interdisciplinary Centre of Natural Products, University of Szeged, 6720 Szeged, Eötvös u. 6, Hungary; hohmann.judit@szte.hu
* Correspondence: vasas.andrea@szte.hu; Tel.: +36 62546451 A.V.; lakatos.lorant @ brc.hu, L.L.

Table of content

Figure S1. ^{1}H NMR spectrum of compound **1** (500 MHz, in $CDCl_3$) 3

Figure S2. ^{13}C JMOD NMR spectrum of compound **1** (125 MHz, in $CDCl_3$) 3

Figure S3. ^{1}H NMR spectrum of compound **2** (500 MHz, in $CDCl_3$) 4

Figure S4. ^{13}C JMOD NMR spectrum of compound **2** (125 MHz, in $CDCl_3$) 4

Figure S5. ^{1}H NMR spectrum of compound **3** (500 MHz, in $CDCl_3$) 5

Figure S6. ^{13}C JMOD NMR spectrum of compound **3** (125 MHz, in $CDCl_3$) 5

Figure S7. ^{1}H NMR spectrum of the mixture of compounds **4** and **5** (500 MHz, in $CDCl_3$)...... 6

Figure S8. ^{13}C JMOD NMR spectrum of the mixture of compounds **4** and **5** (125 MHz, in $CDCl_3$) 6

Figure S9. ^{1}H NMR spectrum of compound **6** (500 MHz, in $CDCl_3$) 7

Figure S10. ^{13}C JMOD NMR spectrum of compound **6** (125 MHz, in $CDCl_3$) 7

Figure S11. ^{1}H NMR spectrum of compound **7** (500 MHz, in $CDCl_3$) 8

Figure S12. ^{13}C JMOD NMR spectrum of compound **7** (125 MHz, in $CDCl_3$) 8

Figure S13. ^{1}H NMR spectrum of compound **8** (500 MHz, in $CDCl_3$) 9

Figure S14. ^{13}C JMOD NMR spectrum of compound **8** (125 MHz, in $CDCl_3$) 9

Figure S15. ^{1}H NMR spectrum of compound **9** (500 MHz, in $CDCl_3$) 10

Figure S16. ^{13}C JMOD NMR spectrum of compound **9** (125 MHz, in $CDCl_3$) 10

Figure S17. RTCA (real-time cell analysis) measurement of CI (cell index) values of HPV-Ker cells treated with compounds **1**, **2**, **4+5**, **6** and **9**. Normalized CI * hours values were plotted as a function of concentration of the indicated diterpenoid (logM). 11

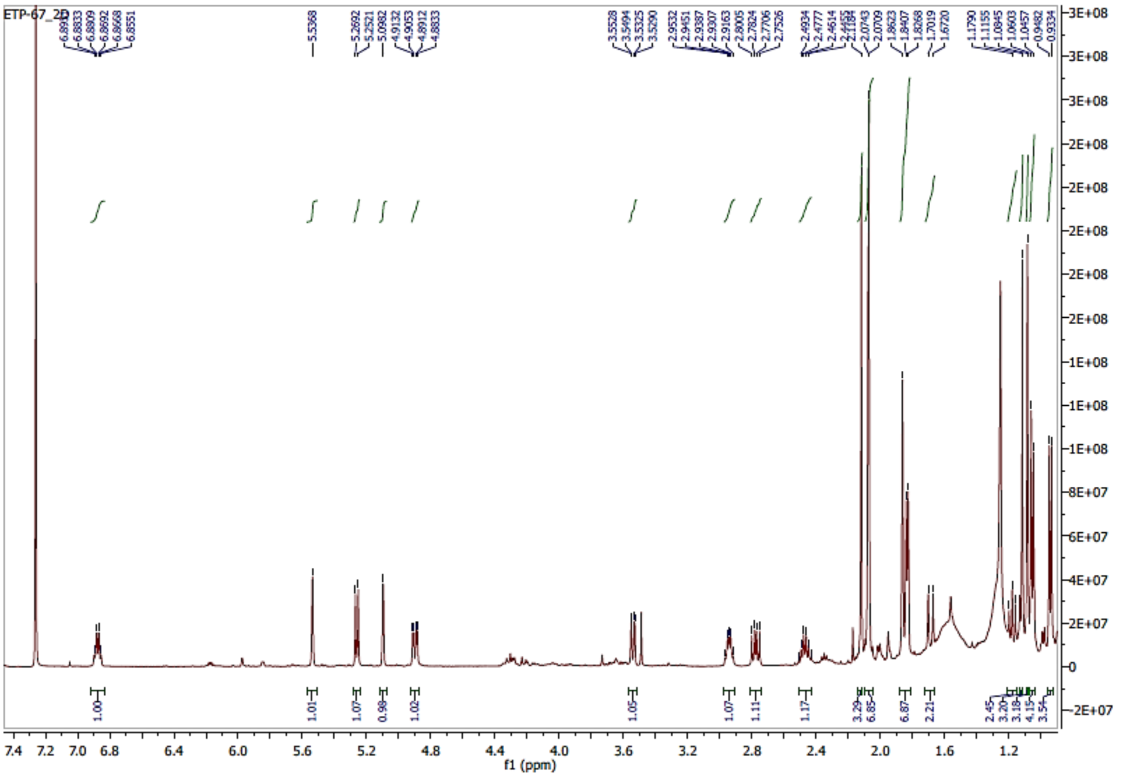

Figure S1. ^{1}H NMR spectrum of compound **1** (500 MHz, in $CDCl_3$)

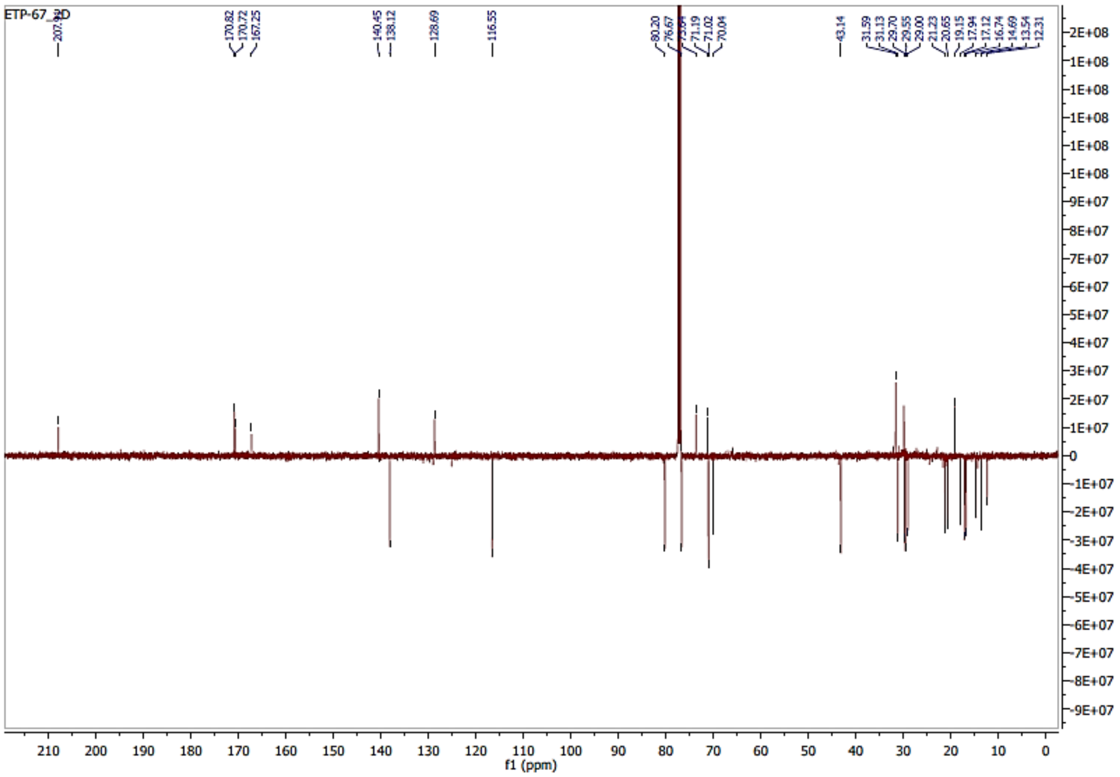

Figure S2. ^{13}C JMOD NMR spectrum of compound **1** (125 MHz, in $CDCl_3$)

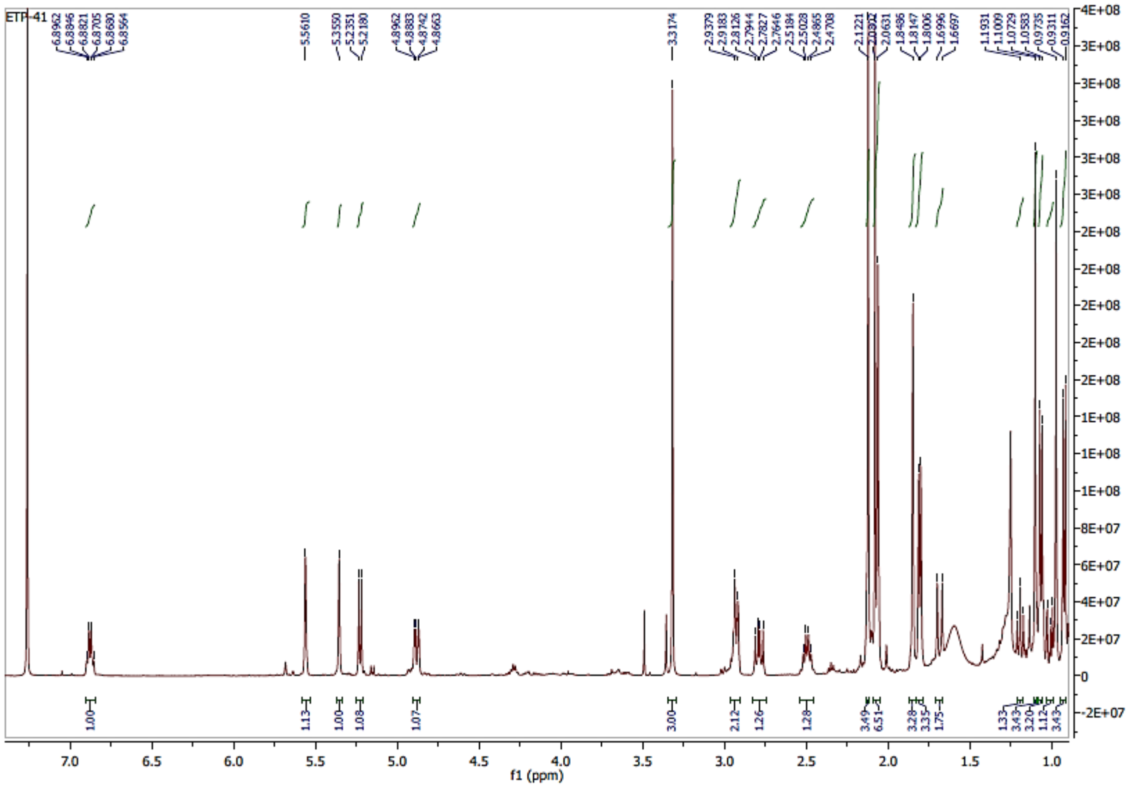

Figure S3. ^{1}H NMR spectrum of compound **2** (500 MHz, in $CDCl_3$)

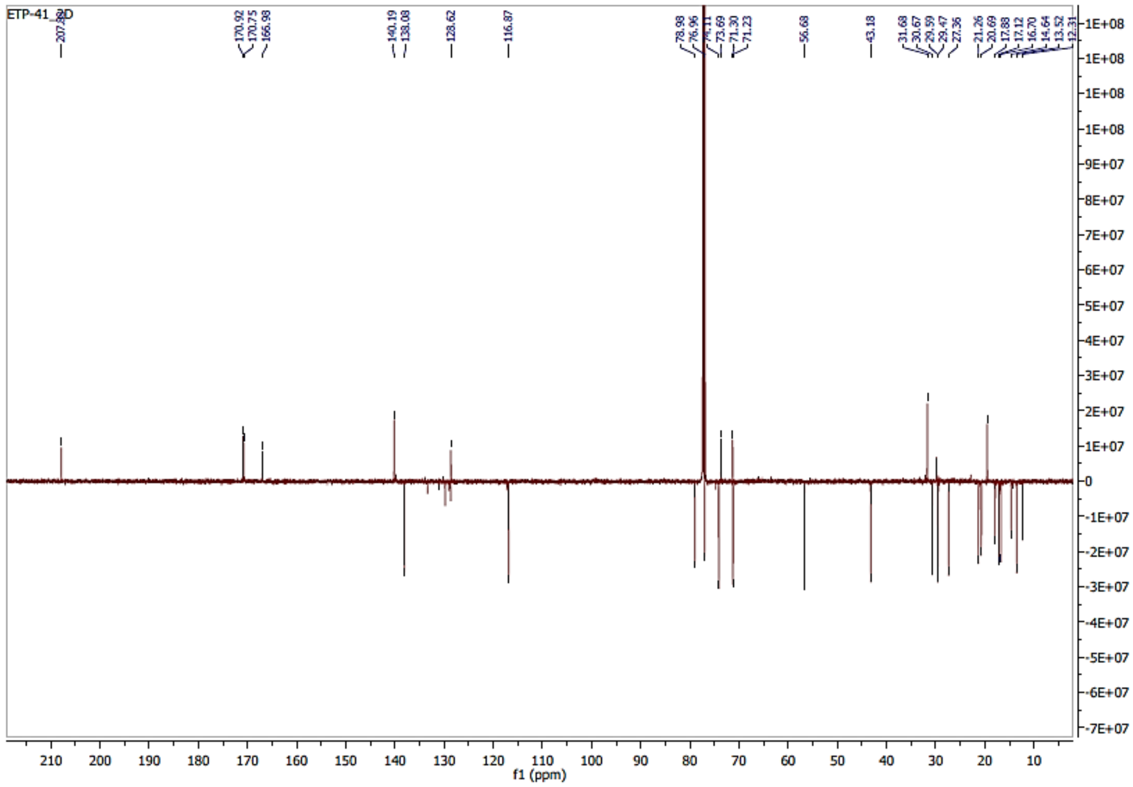

Figure S4. ^{13}C JMOD NMR spectrum of compound **2** (125 MHz, in $CDCl_3$)

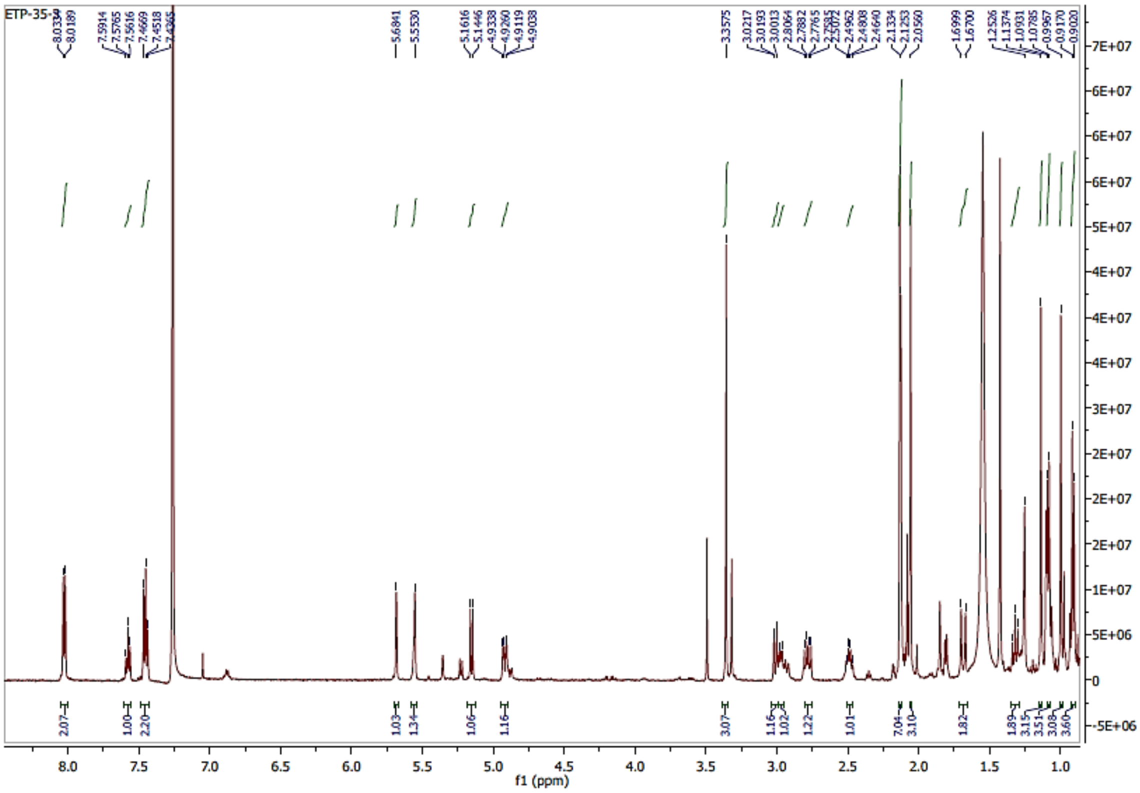

Figure S5. ^{1}H NMR spectrum of compound **3** (500 MHz, in $CDCl_3$)

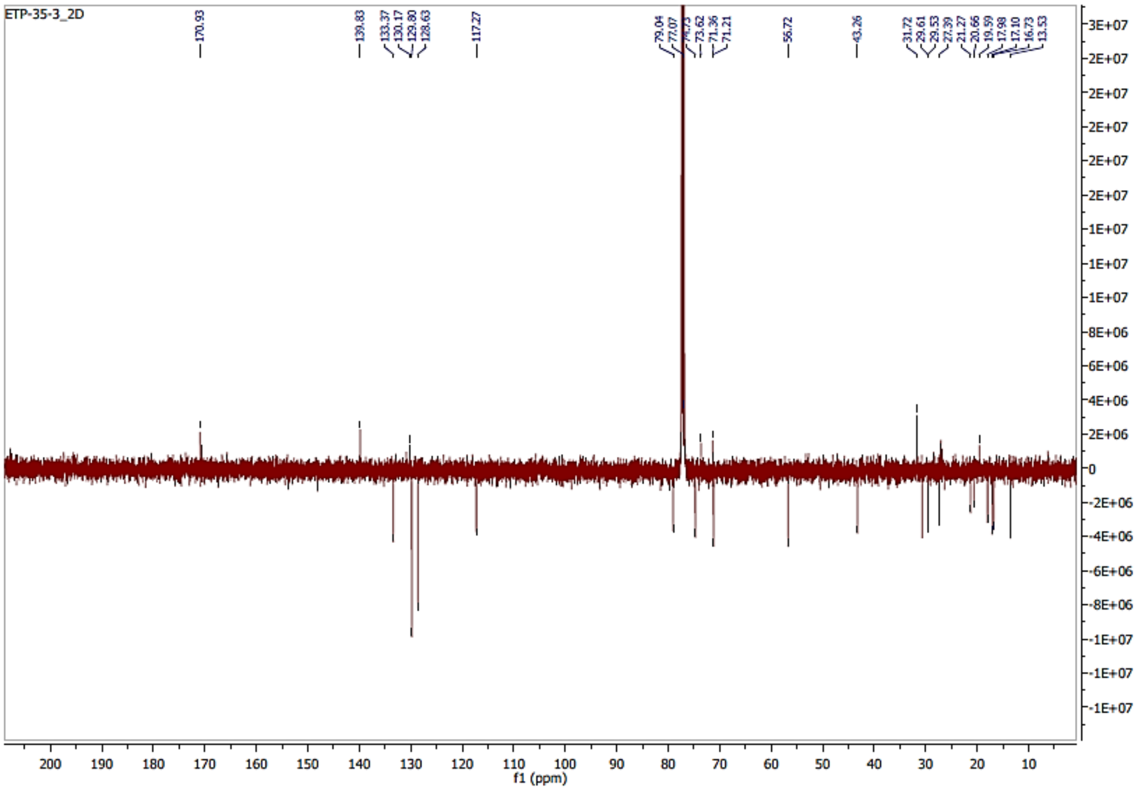

Figure S6. ^{13}C JMOD NMR spectrum of compound **3** (125 MHz, in $CDCl_3$)

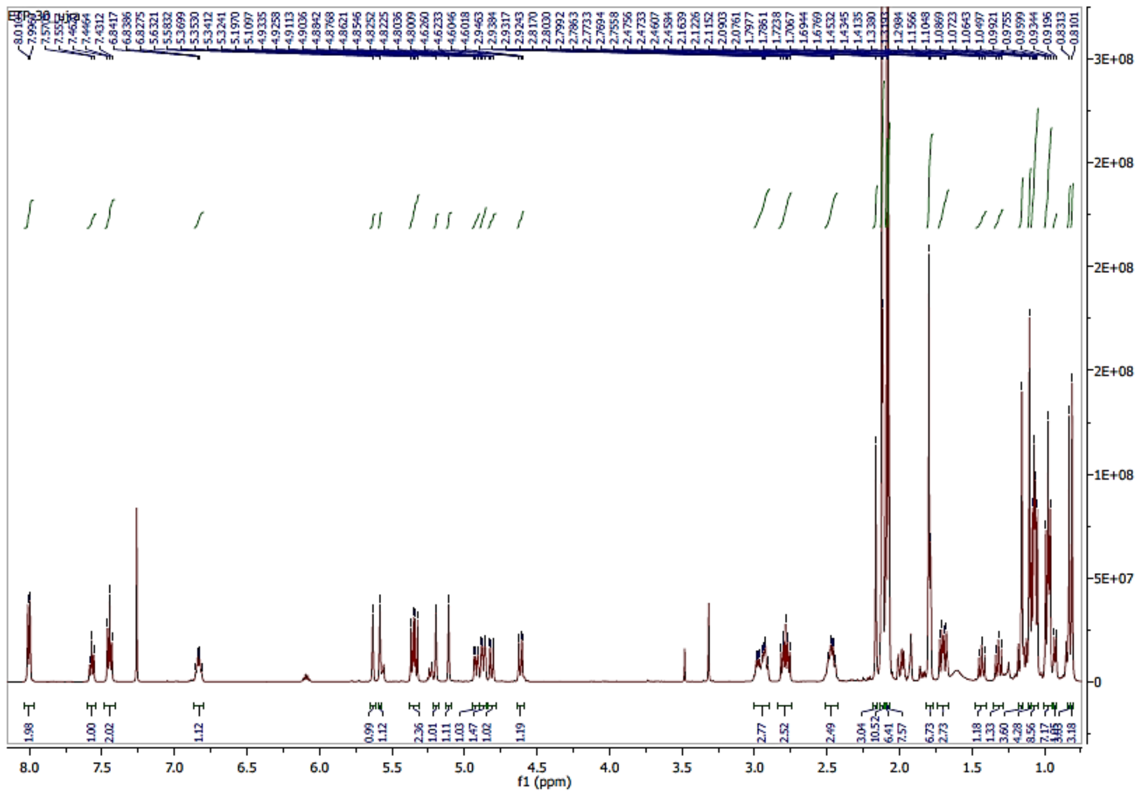

Figure S7. ^{1}H NMR spectrum of the mixture of compounds **4** and **5** (500 MHz, in $CDCl_3$)

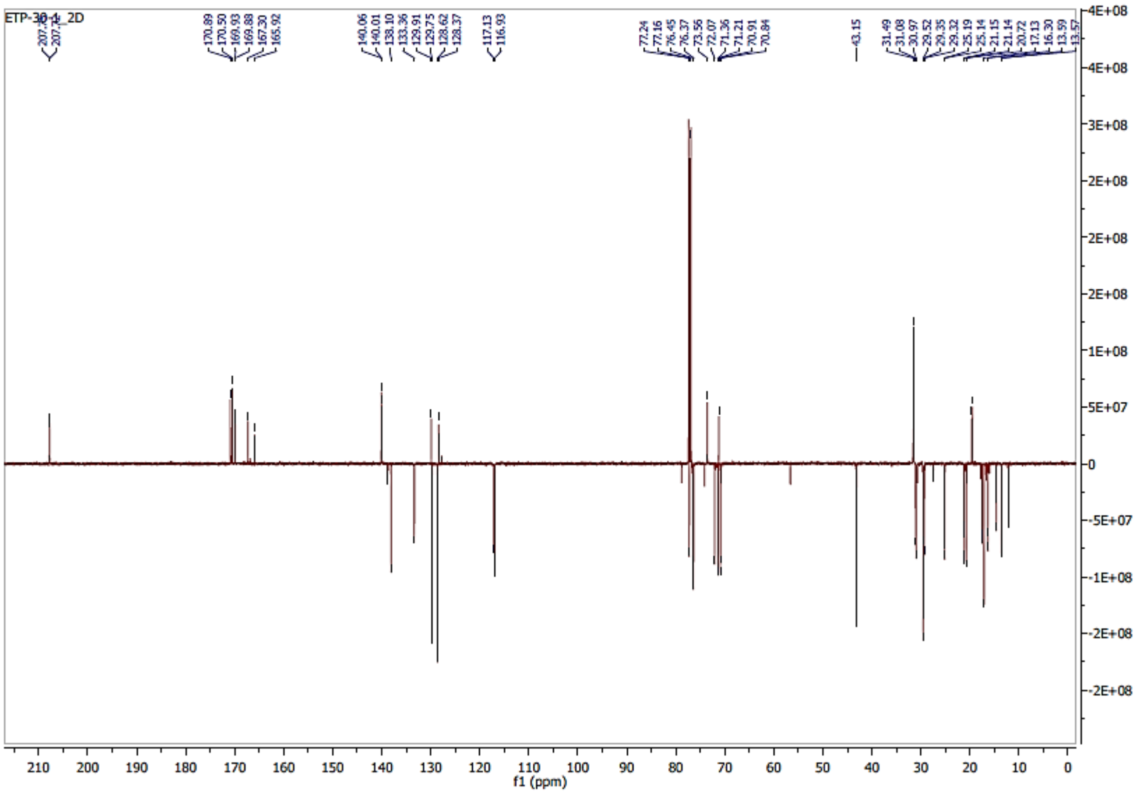

Figure S8. ^{13}C JMOD NMR spectrum of the mixture of compounds **4** and **5** (125 MHz, in $CDCl_3$)

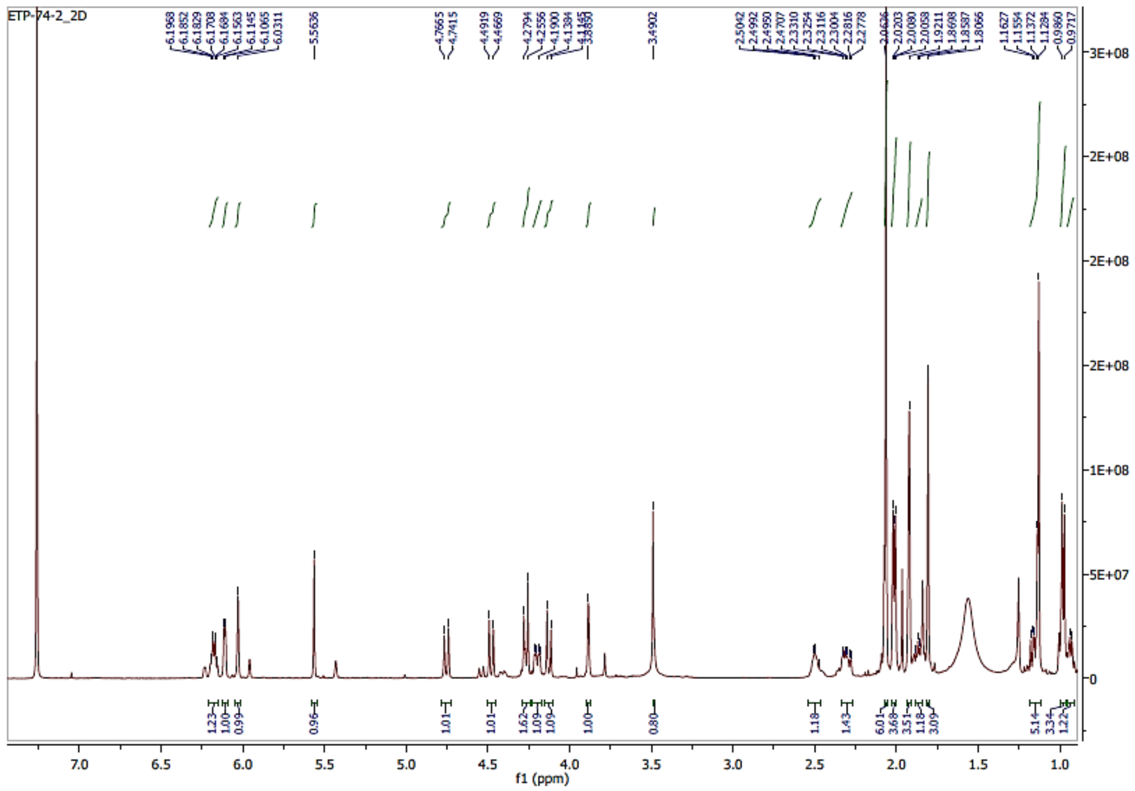

Figure S9. ^{1}H NMR spectrum of compound **6** (500 MHz, in $CDCl_3$)

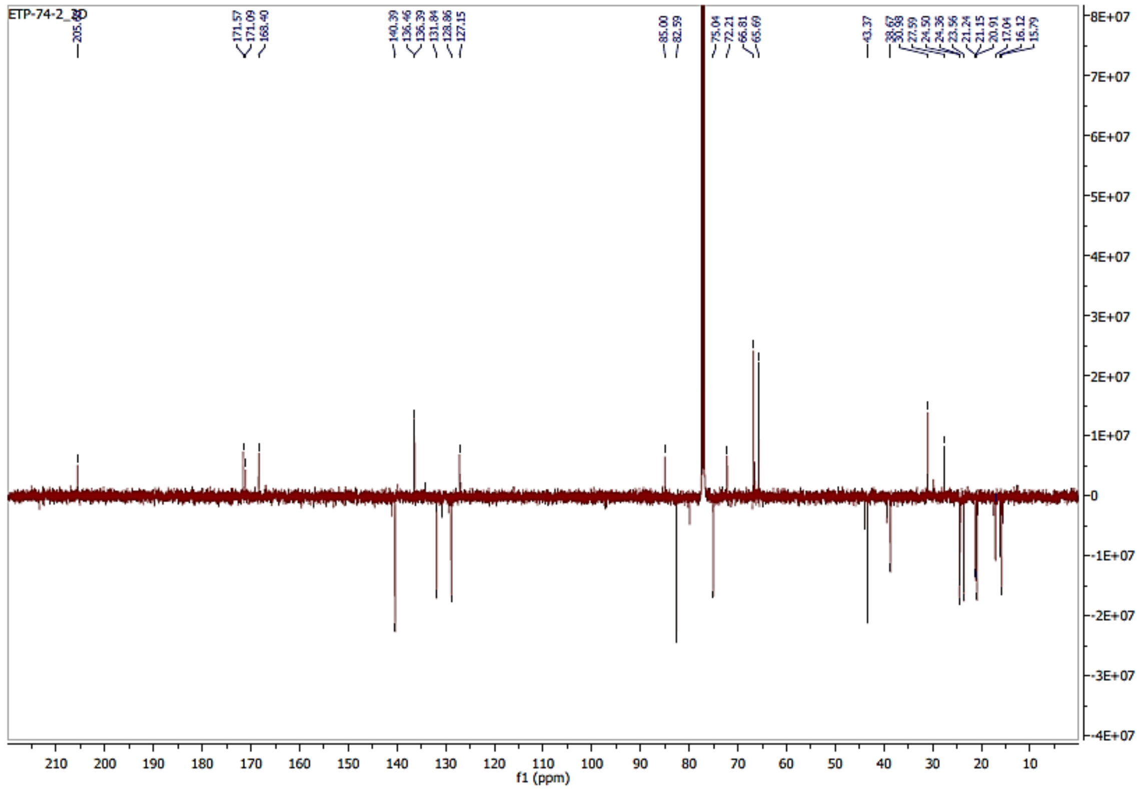

Figure S10. ^{13}C JMOD NMR spectrum of compound **6** (125 MHz, in $CDCl_3$)

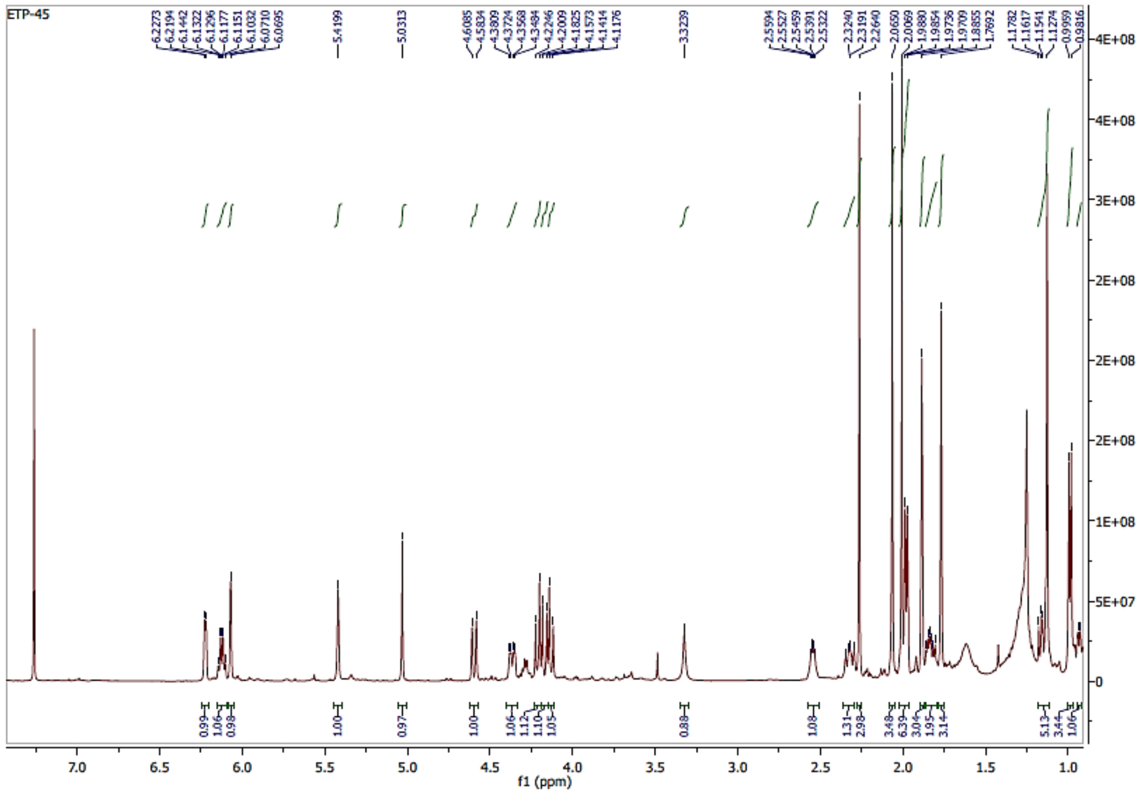

Figure S11. ^{1}H NMR spectrum of compound **7** (500 MHz, in $CDCl_3$)

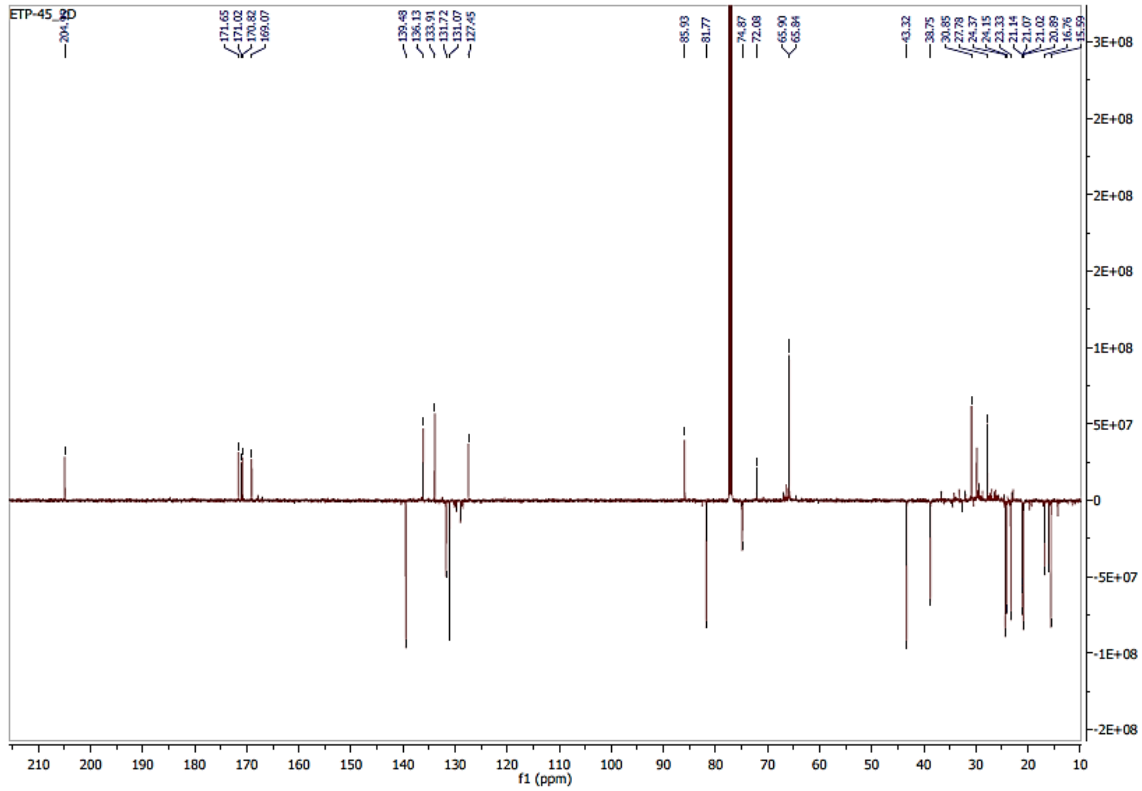

Figure S12. ^{13}C JMOD NMR spectrum of compound **7** (125 MHz, in $CDCl_3$)

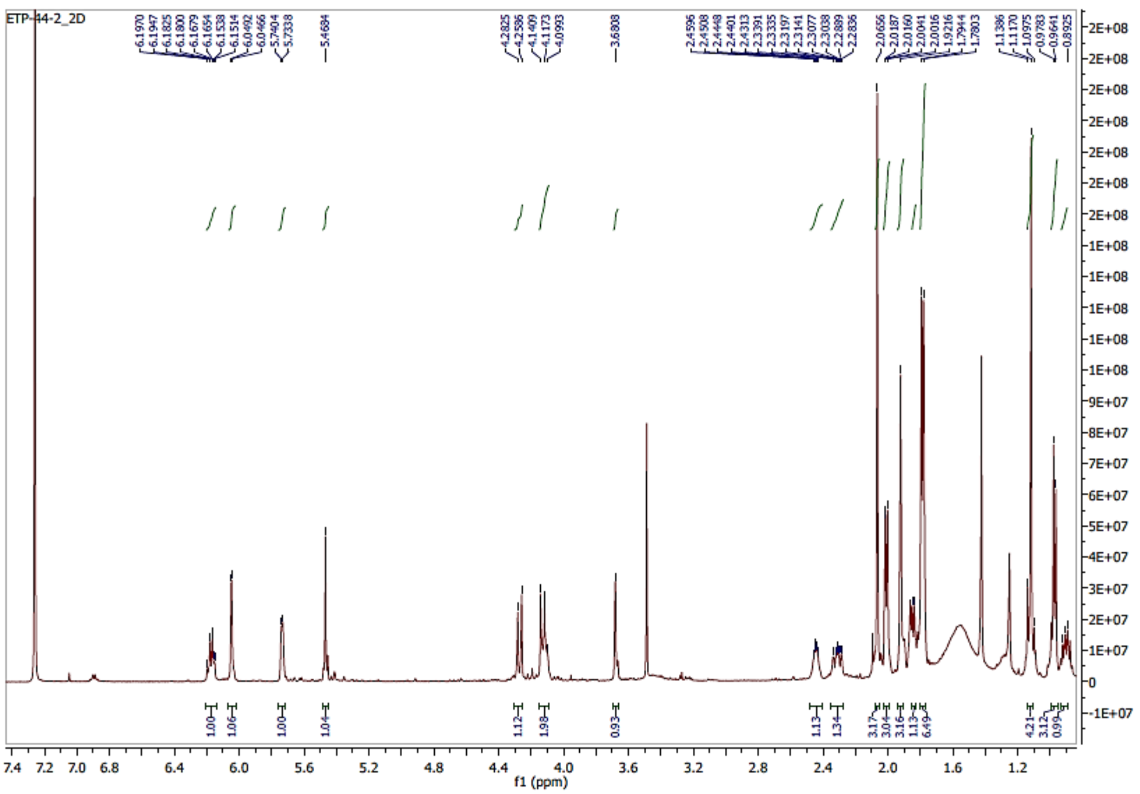

Figure S13. ^{1}H NMR spectrum of compound **8** (500 MHz, in $CDCl_3$)

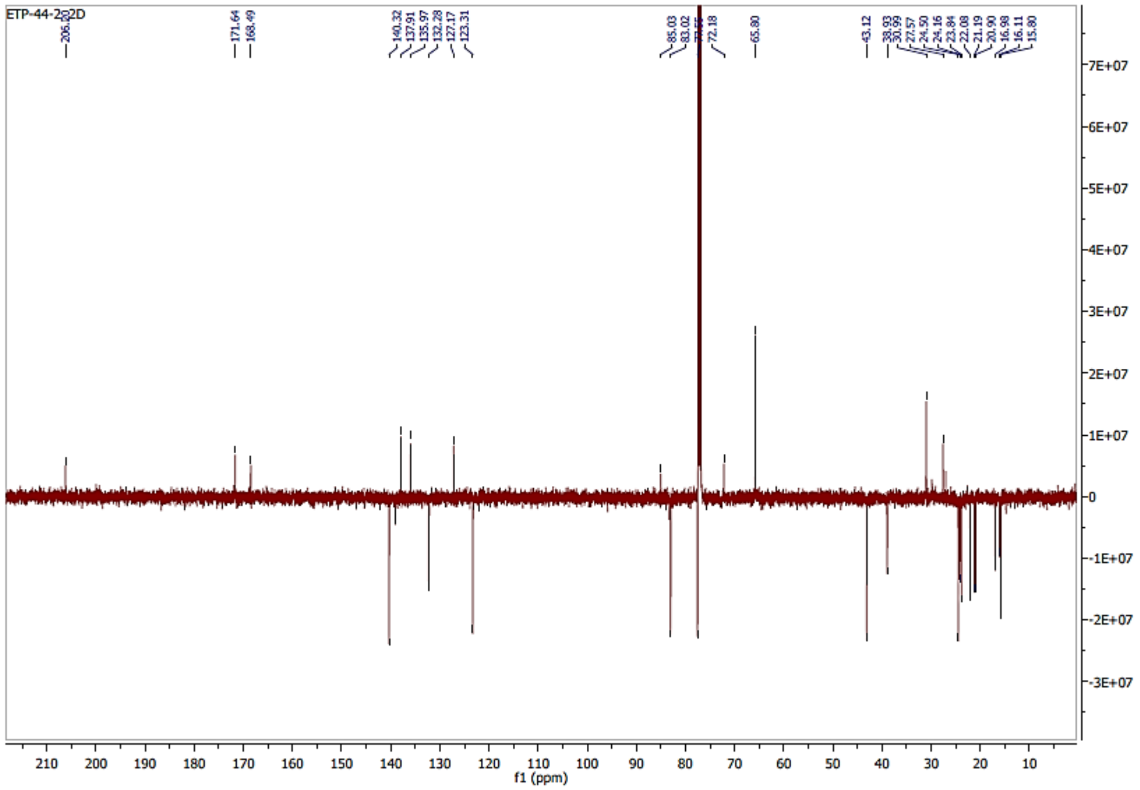

Figure S14. ^{13}C JMOD NMR spectrum of compound **8** (125 MHz, in $CDCl_3$)

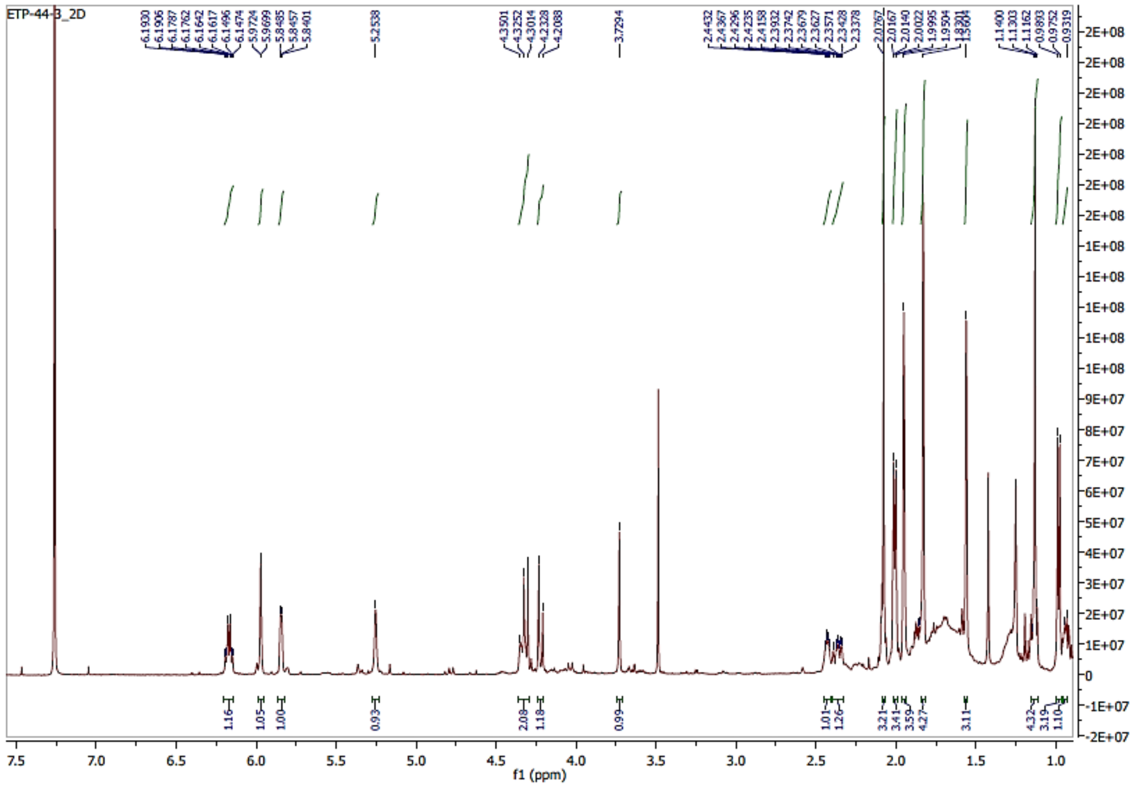

Figure S15. ^{1}H NMR spectrum of compound **9** (500 MHz, in $CDCl_3$)

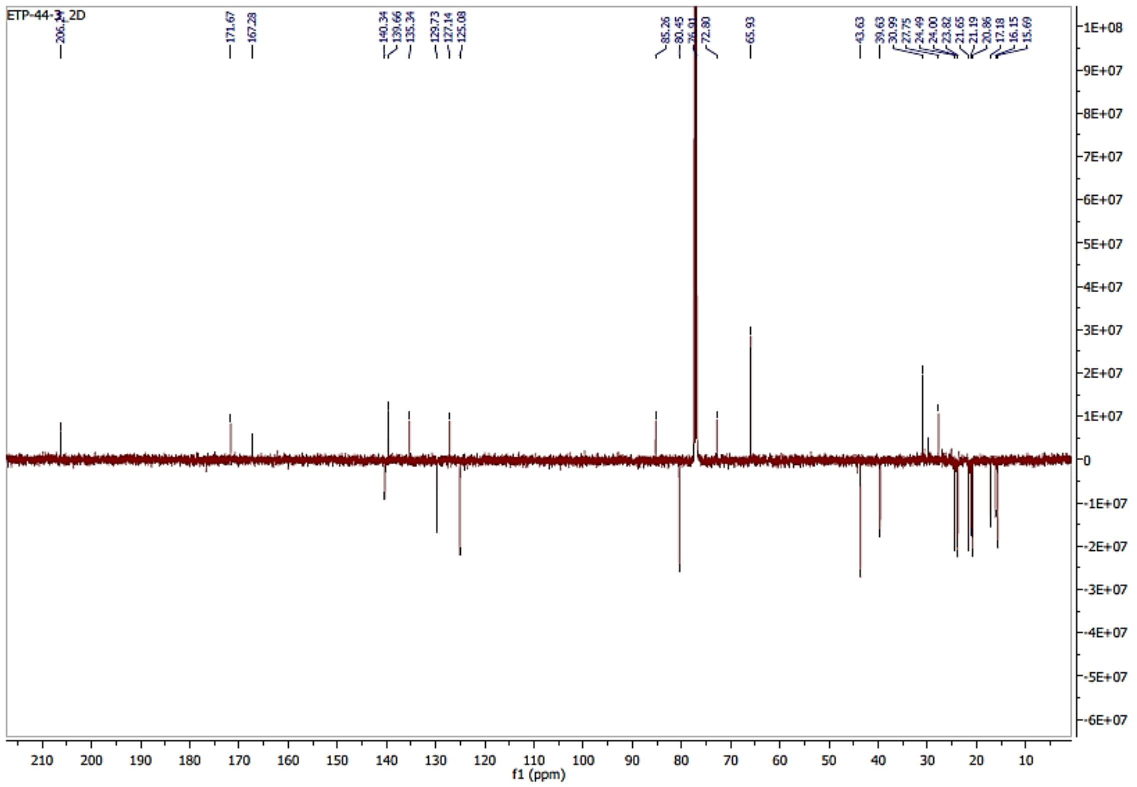

Figure S16. ^{13}C JMOD NMR spectrum of compound **9** (125 MHz, in $CDCl_3$)

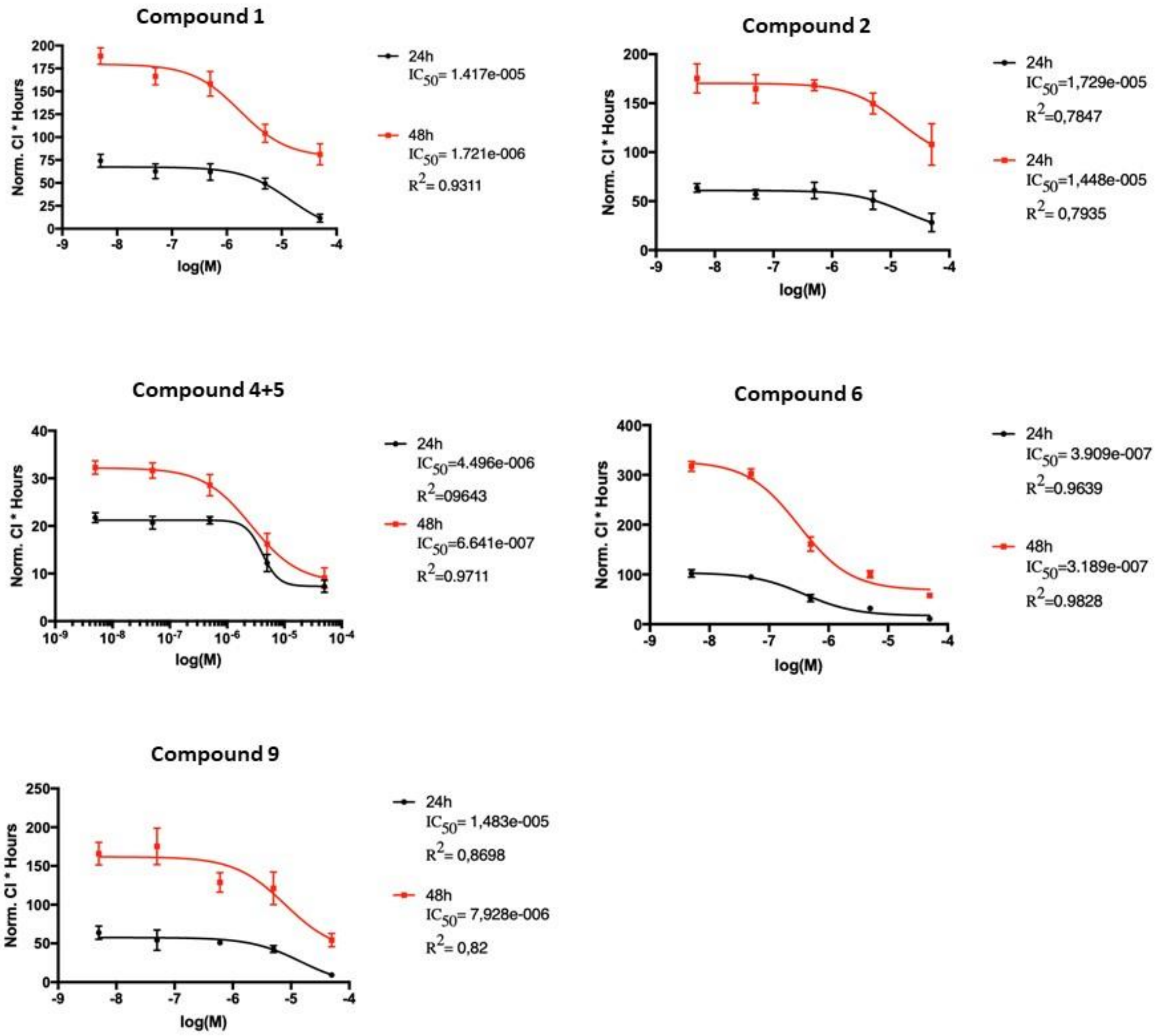

Figure S17. RTCA (real-time cell analysis) measurement of CI (cell index) values of HPV-Ker cells treated with compounds **1**, **2**, **4**+**5**, **6** and **9**. Normalized CI * hours values were plotted as a function of concentration of the indicated diterpenoid (logM).
